# Cajal’s organization of neuronal nucleus revisited

**DOI:** 10.3389/fnana.2025.1724830

**Published:** 2025-12-03

**Authors:** Miguel Lafarga, María T. Berciano, J. Oriol Narcís, Fernando C. Baltanás, Olga Tapia

**Affiliations:** 1 Department of Anatomy and Cell Biology, University of Cantabria, Santander, Spain; 2 “Centro de Investigación en Red Sobre Enfermedades Neurodegenerativas” (CIBERNED), Madrid, Spain; 3 Nash Family Department of Neuroscience, Friedman Brain Institute, Icahn School of Medicine at Mount Sinai, New York, NY, United States; 4 Department of Medical Physiology and Biophysics, Institute of Biomedicine of Seville (IBiS), “Virgen del Rocío” University Hospital, CSIC, University of Seville, Seville, Spain; 5 Department of Basic Medical Sciences, Institute of Biomedical Technologies (ITB), University of La Laguna (ULL), Tenerife, Spain

**Keywords:** Cajal and neuronal nucleus, nuclear condensates, nucleolus, nuclear speckles, Cajal body, transcription factories, nucleoskeleton

## Abstract

In 1906, Cajal was awarded the Nobel Prize in Physiology or Medicine for his pioneering studies on the structure and organization of nerve centers. Notably, in 1910, Cajal published a seminal work in which he described the essential components of the neuronal nucleus, primarily using his reduced silver nitrate procedure. Using modern microscopy techniques, we have identified the current equivalents of the structures originally described by Cajal. These include the “fibrillar center–dense fibrillar component units” of the nucleolus, “nuclear speckles,” “transcription factories,” and “the Cajal body.” Importantly, these structures represent key nuclear compartments involved in the transcription of rDNA and protein-coding genes, pre-rRNA and pre-mRNA processing and spatial genome organization. Most of the nuclear components described by Cajal are now recognized as dynamic “nuclear condensates” assembled through liquid–liquid phase separation mechanisms that depend on various categories of RNA and RNA-binding proteins.

## 1 Introduction

**Figure 1:**
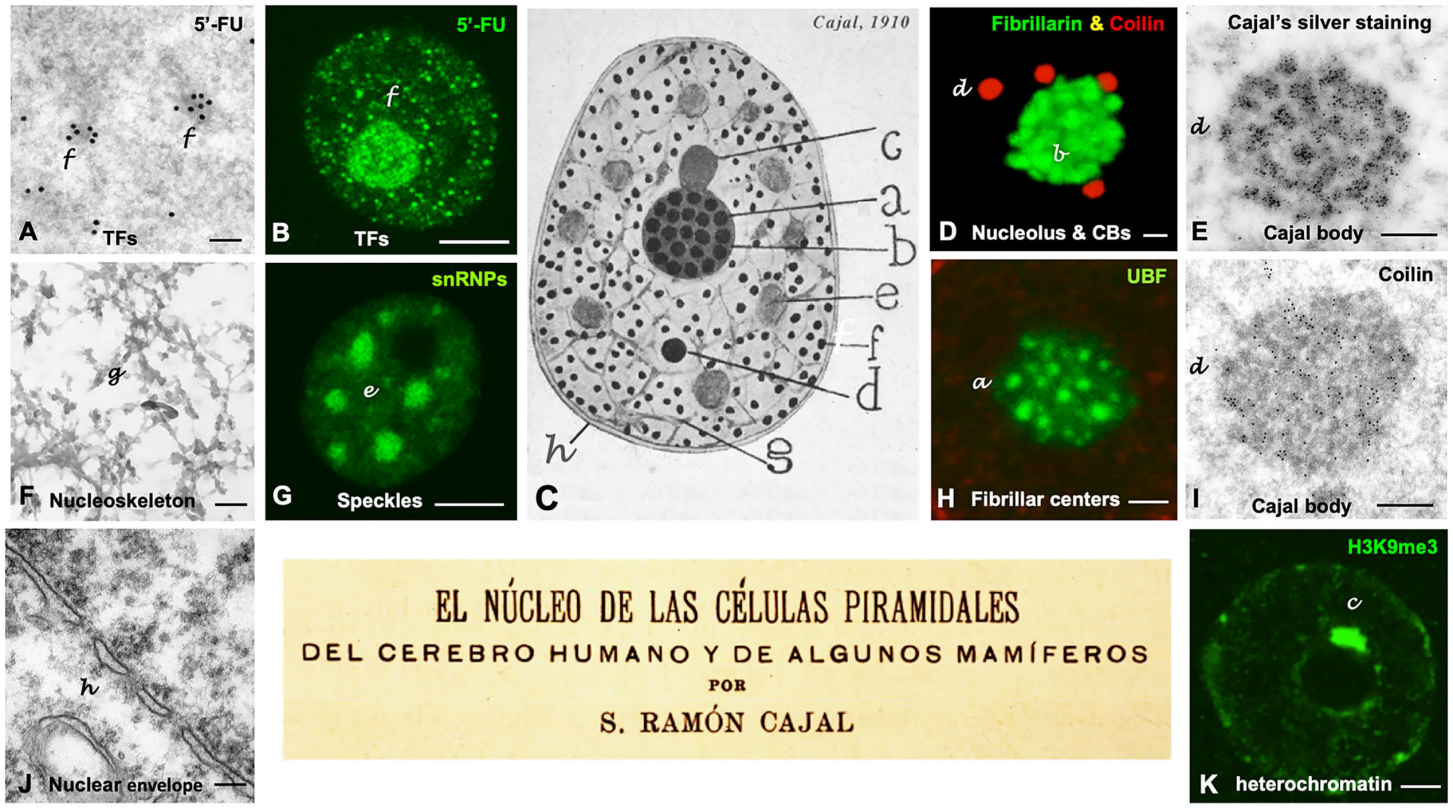
Cajal’s organization of the neuronal nucleus. **(A–K)** Nuclear structures illustrated by Cajal and the equivalent nuclear components identified using modern microscopy techniques in mammalian neurons. Note a dual letter codification in the panels, with the aim of distinguishing between Cajal’s typography (in lowercase and italics) and the modern annotations (regular format). The letters labeling the distinct nuclear structures in the Cajal’s original drawing in panel C are also employed to identify each specific nuclear compartment in the rest of the panels. **(A,B)** Light and electron microscopy *in situ* transcription assay illustrating the incorporation of 5′-FU in neutrophil granules/“transcription factories” (TFs) in control rat sensory ganglion neurons (**A**, reproduced from Lafarga et al., 2017, RNA Biol Figure 1H, with permission from Taylor & Francis; B, reproduced from Casafont et al., 2016, Mol Neurobiol Figure 1B, with permission from Springer Nature). **(C)** Cajal’s original drawing of the neuronal nucleus (From Legado Cajal, Instituto Cajal-CSIC. Madrid, first published in Santiago Ramon y Cajal, 1910, Figure 14). **(D)** Double immunolabeling for fibrillarin (nucleolus, green) and coilin (accessory bodies/“Cajal bodies,” red). Control rat sensory ganglion neuron. **(E)** Electron microscopy cytochemistry using a modification of the Cajal’s silver staining procedure showing silver precipitates specifically decorating the coiled threads of an accessory body/“Cajal body.” Control rat Purkinje neuron (Reproduced from Lafarga et al., 1995, Anat Embryol (Berlin) Figure 11, with permission from Springer-Nature). **(F)** Electron micrograph of a semithin resinless section illustrating the linin framework/“nucleoskeleton.” Control rat sensory ganglion neuron (Reproduced from Lafarga et al., 2009, Chromosoma, Figure 1E, with permission from Springer Nature). **(G)** Confocal microscopy image of hyaline grumes/“nuclear speckles” immunostained for spliceosomal snRNPs. Control rat sensory ganglion neuron (Reproduced from Lafarga et al., 2009, Chromosoma Figure 2d, with permission from Springer Nature). **(H)** Argyrophilic nucleolar spherules/“Fibrillar centers” immunolabeled for the nucleolar transcription factor UBF. Control mouse spinal cord motor neuron (Reproduced from Lafarga et al., 2017, RNA Biol Figure 1D, with permission of Taylor & Francis). **(I)** Immunogold electron microscopy for the detection of coilin in the coiled threads of an accessory body/“Cajal body.” Control rat sensory ganglion neuron (Reproduced from Lafarga et al., 2017, RNA Biol Figure 3F, with permission from Taylor & Francis). **(J)** Electron micrograph of the double membrane of the nuclear envelope and the perinuclear space. Control mouse spinal cord motor neuron. (K) Levi basophilic grume/“perinucleolar heterochromatin” immunolabeled for the histone H3K9me3. Control mouse spinal cord motor neuron (Reproduced from Lafarga et al., 2017, RNA Biol Figure 1B, with permission from Taylor & Francis). Scale bars: A, 250 nm; B, 4 μm; D, 1 μm; E, 250 nm; F, 300 nm; G, 4 μm; H, 800 nm; I, 300 nm; J, 180 nm; K, 1 μm.

Cajal is regarded as the father of modern neuroscience for his work on the neuronal architecture and synaptic connectivity of the nervous system, a contribution recognized with the Nobel Prize in Physiology or Medicine in 1906. A few years later, in 1910, Cajal published the article “The Nucleus of Pyramidal Cells in the Human Brain and Some Mammals,” in which he provided a very accurate and reliable description of key nuclear structures (Figure 1), based on their distinct silver affinities (Cajal, 1910; Lafarga et al., 2009, 2017).

In this review, we compare Cajal’s observations of nuclear components -particularly those obtained with his reduced silver nitrate procedure (Cajal, 1903), with the present-day equivalents identified using modern microscopy techniques. We focus primarily on the current understanding of four key nuclear components involved in transcription and in rRNA and mRNA processing: (i) nucleolar argyrophilic spherules/“fibrillar center–dense fibrillar component units” of the nucleolus, (ii) hyaline grumes/“nuclear speckles,” (iii) neutrophil granules/ “transcription factories,” and (iv) the accessory body/“Cajal body” (Figure 1). Three of these nuclear structures -the nucleolus, nuclear speckles, and Cajal bodies (CBs)- are highly dynamic “nuclear bodies.” Their number, size, structure, and spatial organization are influenced by cellular activity and various pathogenic mechanisms associated with neurological and other diseases (Baltanás et al., 2011b; Mao et al., 2011; Staněk and Fox, 2017; Tapia et al., 2017; Szczepankiewicz et al., 2024; Wu et al., 2024a). Notably, the assembly and functional roles of these nuclear bodies contribute to the regulation of the genome’s spatial organization (Shan et al., 2024). While the focus of this review is centered upon the delineation of four nuclear structures defined by Cajal’s investigations, it is essential to acknowledge that the ensuing years have yielded the characterization of a substantial array of other nuclear subcompartments, such as Promyelocytic Leukemia (PML) bodies, polycomb bodies, clastosomes, paraspeckles or anisosomes, which are implicated in the stress response, neurodegenerative diseases and cancer reviewed in (Lafarga et al., 2002; McCluggage and Fox, 2021; Xu et al., 2021; Yu et al., 2021; Abou-Ghali and Lallemand-Breitenbach, 2024).

It is important to note that most of the nuclear structures Cajal observed are membrane-less organelles. These are now commonly referred to as “nuclear condensates” due to their ability to concentrate proteins and nucleic acids in distinct nuclear domains. The formation of nuclear condensates represents a fundamental mechanism of nuclear organization, as they participate in essential cellular functions such as RNA metabolism, signal transduction, gene regulation, and the DNA damage response (Banani et al., 2017; Lyon et al., 2021; Yu et al., 2021). Nuclear condensates form through condensation and phase separation driven by multivalent macromolecular interactions, particularly those involving various categories of RNAs and RNA-binding proteins containing intrinsically disordered regions (IDRs) (Hyman et al., 2014; Laflamme and Mekhail, 2020; Lyon et al., 2021; Hirose et al., 2023). Recent studies have shown that the presence of IDRs in nuclear condensate proteins facilitates the multivalent interactions underlying phase separation (reviewed in Dogra and Kriwacki, 2025). However, recent reports have proposed alternative mechanisms and highlighted challenges to the phase separation model, particularly with respect to “transcription factories” and “perinucleolar heterochromatin” (reviewed in Peng and Weber, 2019; Laflamme and Mekhail, 2020; Stortz et al., 2024). A major criticism is that most *in vitro* observations of condensate formation cannot be directly extrapolated to native proteins under physiological conditions (Stortz et al., 2024).

Finally, it is worth emphasizing that Cajal conducted his investigations of nuclear structures in the pyramidal neurons of the cerebral cortex (DeFelipe and Fariñas, 1992). The diverse phenotypes of these neurons, characterized by variations in cell body size, synaptic activity, and transcriptional demand, provided a useful model for correlating global neuronal activity with nuclear organization (Pena et al., 2001; Lafarga et al., 2017). Due to the methodological limitations of the early 20th century, Cajal’s study of nuclear components was primarily descriptive. Nonetheless, he showed remarkable foresight by anticipating the dynamic nature of several nuclear structures, which he inferred by comparing their organization across different pyramidal neuron types. For example, as will be discussed later, Cajal established a positive correlation between the size of pyramidal neurons -a parameter dependent on global transcription rate and protein synthesis activity- and the number of argyrophilic spherules/“fibrillar center–dense fibrillar component units” of the nucleolus, which are involved in ribosome biogenesis.

## 2 Nucleolar argyrophilic spherules/“fibrillar center–dense fibrillar component (FC–DFC) units”

**Figure 2:**
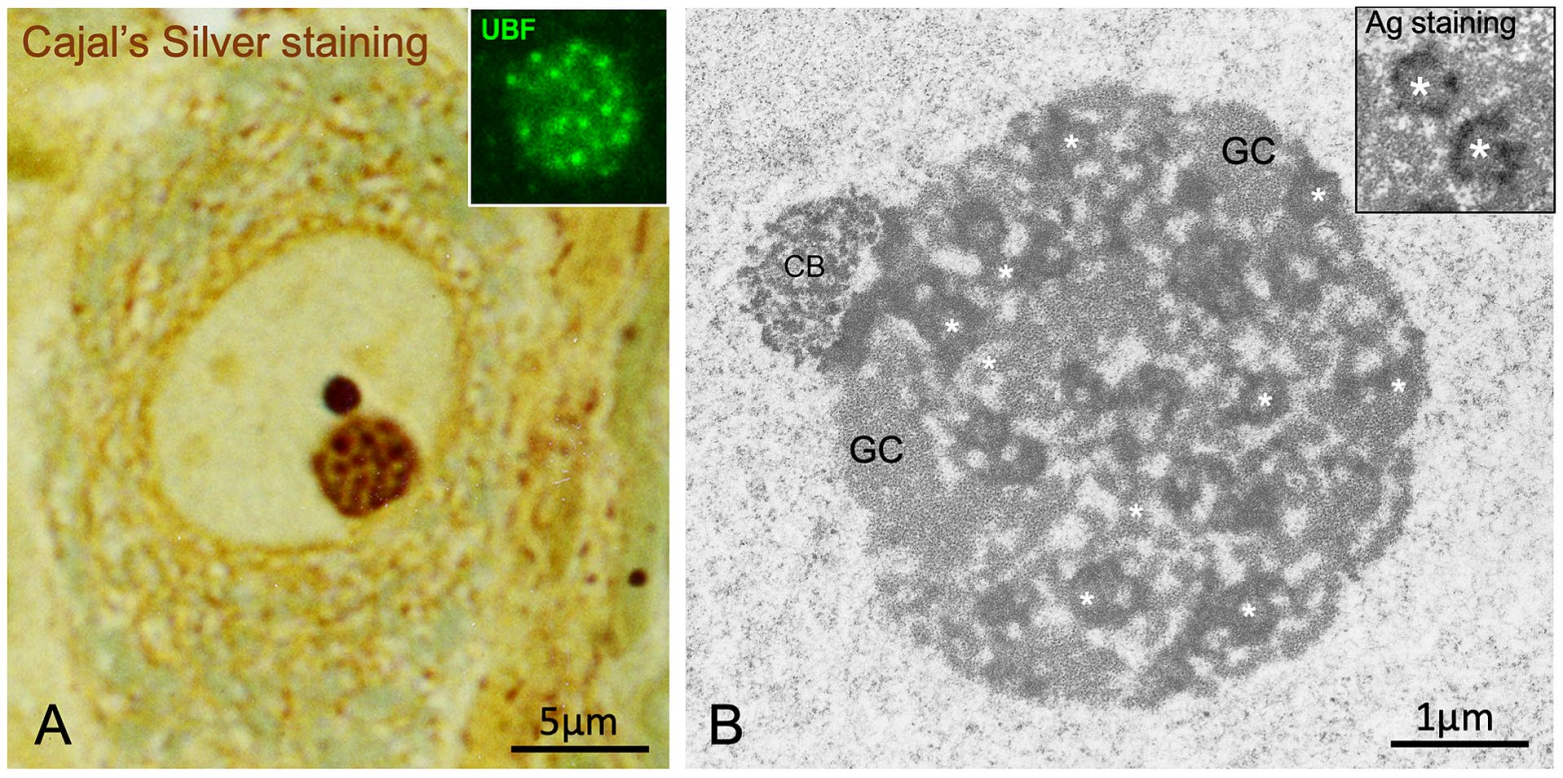
Argyrophilic nucleolar spherules/“FC-DFC units”. **(A)** Cajal’s silver staining of argyrophilic nucleolar spherules/“FC-DFC units of the nucleolus” embedded in a ground substance with low affinity for silver in a large rat sensory ganglion neuron. Note a prominent, sharply defined, and intensely stained accessory body/“Cajal body” attached to the nucleolus (Reproduced from Lafarga et al. J Neuroscience Methods, 1986, with permission from Elsevier). Inset: Detail of a nucleolus with numerous small FCs immunolabeled for the nucleolar transcription factor UBF. Control mouse sensory ganglion neuron (Reproduced from Tapia et al. Neurobiol Dis 2017, Figure 5A inset, with permission from Elsevier). **(B)** Electron micrograph of a nucleolus from a control rat sensory ganglion neuron composed of numerous smaller-sized argyrophilic nucleolar spherules/“FC/DFC units” (asterisks) and associated granular component (GC). Note the Cajal body (CB) associated with the nucleolus. (Reproduced from Palanca et al. Biochim Biophys Acta 2014, Figure 2C, with permission from Elsevier). 2B Inset: Ultrastructural AgNOR staining of argyrophilic nucleolar spherules/“FC-DFC units” (asterisks) in a TG cell (a cell line from human fallopian tube cancer) (Reproduced from Penzo et al., Cells 2019, reproduction partial of the Figure 1B with permission from MDPI).

Cajal described the neuronal nucleolus as consisting of two main components: closely packed argyrophilic spherules and a ground substance that was refractory to silver staining but showed a strong affinity for basic anilines (Figure 1C). He provided the first detailed description of the structure, size (0.25–0.30 μm in diameter), and number of argyrophilic spherules (Figures 1C,2A). It is now well established that in large mammalian neurons, the fine structure of the nucleolus is composed of numerous smaller “fibrillar center (FC)–dense fibrillar component (DFC) units,” surrounded by the “granular component” (Figure 2B) (Pena et al., 2001; Baltanás et al., 2011b; Palanca et al., 2014). The organization of argyrophilic nucleolar spherules closely corresponds to nucleolar spots immunolabeled for the nucleolar transcription factor upstream binding protein (UBF), a marker of FCs (Figure 2A, inset) (Hernandez-Verdun et al., 2010; Tapia et al., 2017).

Electron microscopy cytochemistry for silver-affinity proteins has revealed that argyrophilic spherules correspond to interphase “nucleolar organizer regions” (NORs), where transcriptionally active rDNA, encoding ribosomal RNAs (rRNAs) 28S, 18S and 5.8S, is located (reviewed in Penzo et al., 2019). NOR silver staining highlights two ultrastructural nucleolar components: the FC and the associated DFC (Figure 2B, inset) (Lafarga et al., 1995; Penzo et al., 2019), which together constitute FC–DFC functional units where rRNA synthesis and early rRNA processing occur (Hernandez-Verdun et al., 2010; Lafontaine et al., 2021; Shan et al., 2024; Pan et al., 2025). Transcription of rRNA genes takes place at the FC/DFC border. Two classical protein markers of the FC are RPA194 -a subunit of the RNA polymerase I (RNA pol I) complex- and the nucleolar transcription factor UBF. The DFC plays a key role in the initial processing steps of pre-rRNAs. It contains newly synthesized pre-rRNAs and associated proteins such as fibrillarin, Nop56, and Nop58, which collectively form a small nucleolar ribonucleoprotein (snoRNP) complex essential for proper rRNA processing. Silver-affinity proteins of NORs include the largest subunit of RNA polymerase I, UBF, and nucleolin (Penzo et al., 2019). The equivalent of the nucleolar ground substance described by Cajal (1910) is the “granular component” at the ultrastructural level, which lacks silver affinity and represents the nucleolar substructure where pre-ribosomal particles are assembled (Figures 1C,2B).

Another remarkable observation by Cajal was the positive correlation between the size of pyramidal neurons and the number of argyrophilic spherules: from 4 to 8 in small neurons and from 24 to 36 in large ones (Cajal, 1910). At present, the close relationship between the number of FCs and the size of both the nucleolus and the neuronal body has been confirmed in human sensory ganglion neurons (Berciano et al., 2007). Because argyrophilic spherules correspond to FC–DFC units, Cajal’s findings suggest a positive correlation between argyrophilic spherule number and both rDNA transcription rate and ribosome biogenesis in neurons. Similarly, Cajal reported that the number of nucleoli in pyramidal neurons inversely correlated with cell body size, resulting in mononucleolated large neurons, whereas small neurons tended to be multinucleolated. This nucleolar behavior, confirmed in other neuronal populations (Pena et al., 2001; Berciano et al., 2007), indicates that in maturing neurons, the fusion of NORs into single or multiple nucleoli is related to neuronal size and global transcriptional activity (Pena et al., 2001).

Recent studies support the view that the nucleolus is a multilayered nuclear condensate whose assembly through phase separation facilitates the sequential steps of ribosome biogenesis (Lafontaine et al., 2021). Phase separation gives rise to several nucleolar subcompartments, including multiple microphases of argyrophilic spherules/“FC–DFC units,” through nucleolar protein–protein and protein–RNA interactions (Yamamoto et al., 2023).

Beyond its canonical function in ribosome biogenesis, the nucleolus is recognized as an essential sensor of cellular stress (Boulon et al., 2010). Accordingly, nucleolar stress, defined as the disruption of nucleolar integrity and the subsequent inhibition of rRNA synthesis and processing, can severely disrupt cellular homeostasis and ultimately lead to neuronal death (González-Arzola, 2024; Sirozh et al., 2024; Wu et al., 2024b). Nucleolar stress has been implicated in the pathogenesis of amyotrophic lateral sclerosis (ALS) and frontotemporal lobar degeneration (FTD), particularly in cases associated with GGGGCC hexanucleotide repeat expansions in the C9ORF72 gene. For example, several studies have shown that nucleolar stress precedes the cytoplasmic mislocalization of TDP-43 in motor neurons of both C9ORF72-associated and sporadic ALS patients (Aladesuyi Arogundade et al., 2021). A recent study demonstrated that nucleolar stress induced by arginine-rich peptides leads to a widespread accumulation of ribosome-free ribosomal proteins. This accumulation is toxic to cells and represents a common outcome in response to nucleolar stress (Sirozh et al., 2024).

## 3 Hyaline grumes/“nuclear speckles”

**Figure 3:**
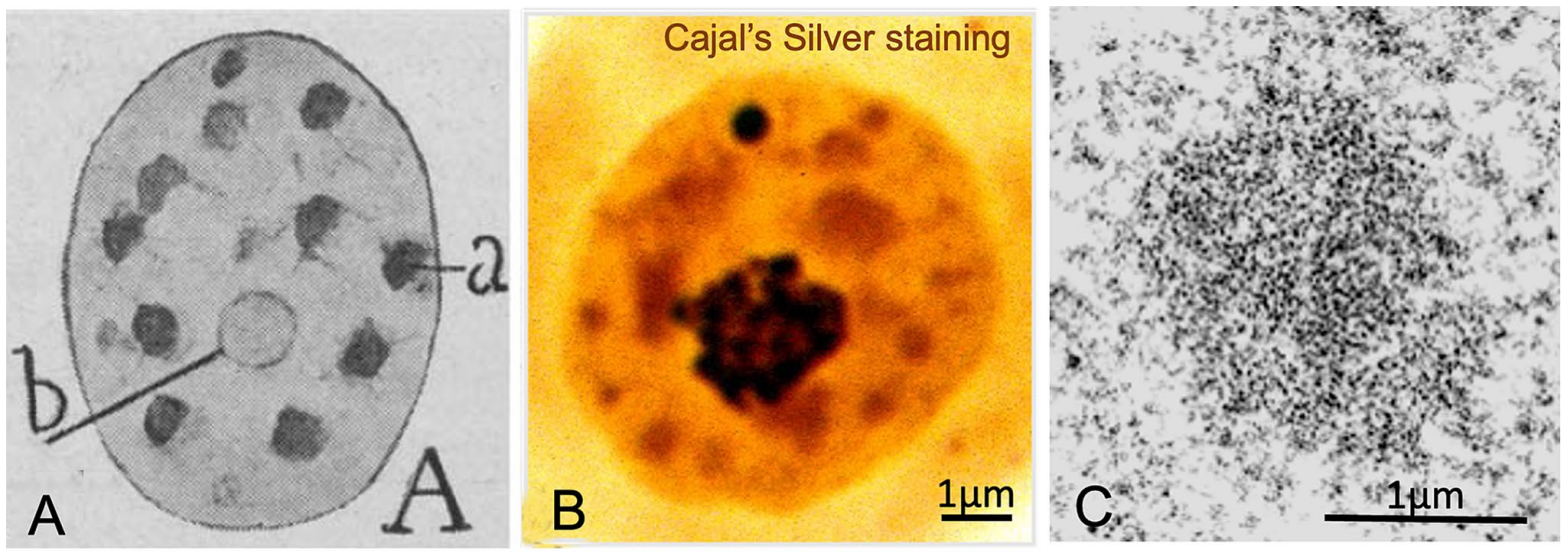
Hyaline grumes/“nuclear speckles”. **(A)** Cajal’s original drawing of hyaline grumes/“nuclear speckles” in a pyramidal neuron. The typography of Cajal’s lettering indicates: (a) hyaline grumes/“nuclear speckles” and (b) nucleolus (From Legado Cajal, Instituto Cajal-CSIC. Madrid, first published in Santiago Ramon y Cajal, 1910, Figure 11A). **(B)** Cajal’s silver staining of the nucleus from a rat sensory ganglion neuron. Whereas the nucleolus and the accessory body/“Cajal body” exhibit a strong silver affinity (dark staining), hyaline grumes/“nuclear speckles” show a moderate silver affinity (orange-like staining) (Reproduced from Lafarga et al., Chromosoma 2009, Figure 2c, with permission from Springer Nature). **(C)** High magnification electron micrograph of an interchromatin hyaline grume/“interchromatin granule cluster”. Control rat sensory ganglion neuron (Reproduced from Pena et al. J Comp Neurol 2001, Figure 9A, with permission of Wiley-Liss Inc).

Cajal described another key structure closely associated with nuclear architecture and function, the hyaline grume, later referred to as a “nuclear speckle” by Beck in 1961 (Beck, 1961). Using acid aniline staining and a specific reduced silver nitrate procedure with formalin-carbamide fixation, Cajal observed round or irregular, homogeneously stained clumps with a translucent appearance in the nucleoplasm, which he termed hyaline grumes (Figures 1C,3A,B). Their number varied from 6 to 11 in nuclear sections of pyramidal neurons, and their size ranged from 0.3 to 1.5 μm in diameter, although some up to 3 μm were also found in large motor neurons. Unlike the argyrophilic nucleolar spherules and the accessory body, which had a strong affinity for colloidal silver (black staining), the hyaline grumes exhibited lower silver affinity, yielding a yellow or red coloration (Figure 3B). Hyaline grumes typically correspond to “nuclear speckles” immunostained for spliceosomal snRNPs (Figure 1G) or to “interchromatin granule clusters” at the ultrastructural level (Figure 3C) (reviewed in Lamond and Spector, 2003; Faber et al., 2022; Chaturvedi and Belmont, 2024). The adaptation of Cajal’s silver staining procedure for electron microscopy confirmed that the ultrastructural equivalent of hyaline grumes are the “interchromatin granule clusters or nuclear speckles”, which were specifically decorated with silver grains (Lafarga et al., 2009).

Nuclear speckles represent a distinct category of nuclear condensates that concentrate pre-mRNA splicing factors, including spliceosomal U1, U2, U4/U6, and U5 snRNPs, as well as other splicing proteins such as SC35/SRSF2, a classical nuclear speckle marker (Lyon et al., 2021; Hirose et al., 2023; Shan et al., 2024). In particular, SRRM2 and SON proteins, through their IDRs, are essential for the assembly of nuclear speckles as nuclear condensates (Ilık et al., 2020). Nuclear speckles are considered sites of mRNA splicing, aberrant mRNA storage, and intronless transcript retention. Therefore, nuclear speckles are tightly linked to pre-mRNA splicing (reviewed in Galganski et al., 2017; Chen and Belmont, 2019; Bhat et al., 2024; Shan et al., 2024). In fact, it has been proposed that approximately 50% of transcriptionally active genes localize at the periphery of nuclear speckles -the speckle-associated domains (SPADs)- where they can directly access the splicing machinery (Faber et al., 2022; Chaturvedi and Belmont, 2024; Wu et al., 2024a).

Recent findings on nuclear speckle dynamics in mammalian neurons, specifically in hippocampal granule cells, have demonstrated that alterations in transcription and splicing lead to both structural and proteomic remodeling of these nuclear bodies (Szczepankiewicz et al., 2024). Moreover, a proteomic study identified the activity-regulated cytoskeleton-associated protein (ARC) as a regulator of both basal and long-term potentiation (LTP)-associated formation of nuclear speckles in dentate gyrus neurons (Kanhema et al., 2025). Finally, several studies have reported functional links between nuclear speckles and neurological diseases. Specifically, the loss of nuclear speckle integrity has been shown to induce widespread dysfunction in mRNA splicing and consequent neuronal degeneration in C9ORF72-associated FTD/ALS (Wu et al., 2024b). Likewise, abnormal nuclear speckle morphology and cytoplasmic mislocalization of the nuclear speckle scaffold protein SRRM2 have been reported in human tauopathies (McMillan et al., 2021; Dion et al., 2025).

## 4 Neutrophil granules/“transcription factories”

Poorly defined nucleoplasmic dots with moderate affinity for both basic and acidic dyes, known as neutrophil granules, had been observed by several researchers (Marinesco, 1905, 1909; Cajal, 1910). Using a fixation variant (formalin-acetic acid or absolute ethanol) of his silver nitrate procedure, Cajal provided an accurate description of the neutrophil granules (Figure 1C). They appeared as sharply defined dots (0.15–0.18 μm) intensely stained with colloidal silver and distributed throughout the nucleoplasm. Cajal pointed out that these nuclear dots were excluded from the nucleolus, the hyaline grumes/“nuclear speckles,” and the Levi basophilic grume/“perinucleolar heterochromatin” (Figure 1C).

**Figure 4:**
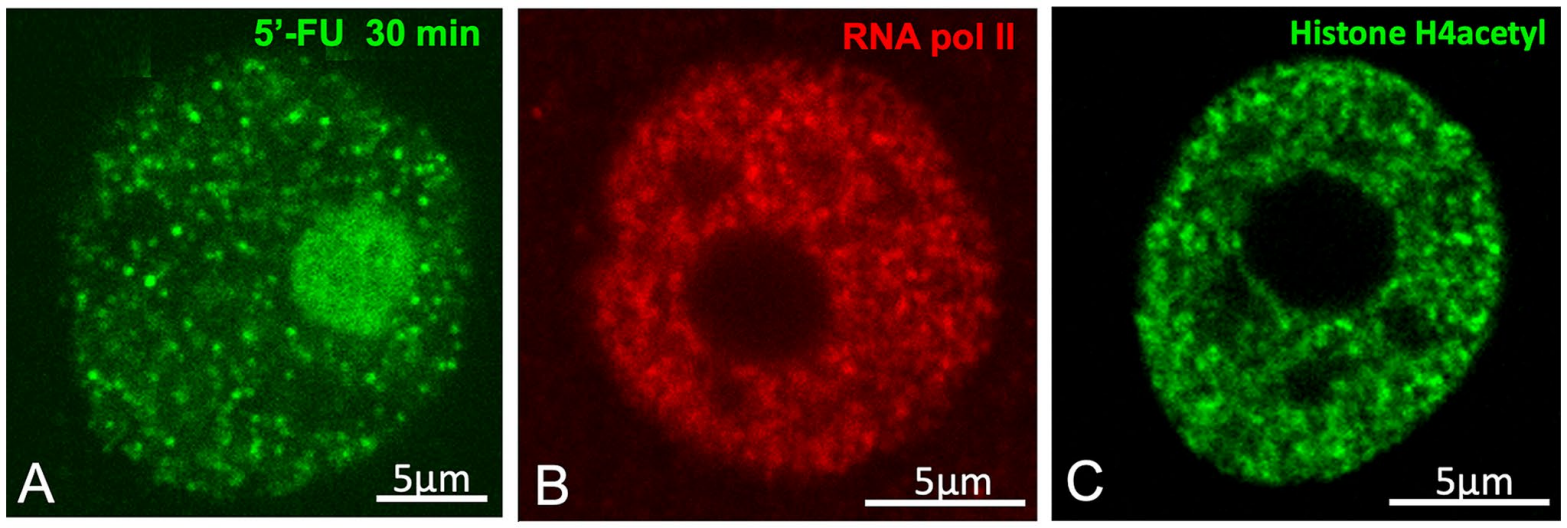
Neutrophil granules/“transcription factories”. **(A)** *In vivo* transcription assay based on the incorporation of 5′-FU into nascent RNAs of a rat sensory ganglion neuron. The nuclear spots of 5′-FU incorporation correspond to the neutrophil granules/“transcription factories”. Hyaline grumes/“nuclear speckles” are visible as negative nucleoplasmic areas of variable size free of transcription factories. **(B)** Immunolabeling for the hyperphosphorylated RNA Pol II at Ser2, illustrating the nuclear foci of high concentrations of active Pol II in the transcription factories. Control rat sensory ganglion neuron (**A,B** from Casafont et al., Mol Neurobiol 2016, Figures 2m, 1A, respectively, with permission from Elsevier). **(C)** Immunolabeling for the acetylated histone H4, with brilliant foci (transcription factories) and negative areas of the nucleolus and nuclear speckles. Control rat sensory ganglion neuron (From Lafarga et al. 2009, Chromosoma Figure. 2E, with permission from Springer Nature).

Importantly, the structural features and spatial distribution of silver-stained neutrophil granules are strikingly similar, or even identical, to the small nuclear foci of 5′-fluorouridine (5´-FU) incorporation into nascent RNA, identified as “transcription factories” (Jackson et al., 1993; Rieder et al., 2012). Our previous studies have also reported these structures in neurons (Figures 1A,B, 4A) (Casafont et al., 2006, 2016; Baltanás et al., 2011a). Moreover, these factories also concentrate active RNA polymerase II hyperphosphorylated at Ser2 and acetylated histone H4 (Figures 4B,C), both of which are protein factors associated with active transcription sites (Cmarko et al., 1999; Rieder et al., 2012; Casafont et al., 2016). According to Cajal’s observations (1910), some factories appear to be associated with the periphery of the hyaline grume/“nuclear speckle,” where they could have direct access to splicing factors, but are excluded from its interior (Figures 1B,C, 4A). Furthermore, Cajal’s measurements of neutrophil granules -ranging from 0.15 μm to 0.18 μm- are remarkably consistent with the current range of transcription factory diameters (45–220 nm), depending on the experimental method used (Rippe and Papantonis, 2025). Using super-resolution microscopy, Castells-Garcia and colleagues identified transcription factory substructures that concentrate nascent RNA, which they termed “RNA nanodomains” (Castells-Garcia et al., 2022).

Transcription factories are nuclear condensates that contain a protein-rich core enriched with clusters of RNA polymerase II/III and transcriptional cofactors that interact with the cis-regulatory elements of active genes. Nascent coding and noncoding RNAs are extruded around this protein core (Sutherland and Bickmore, 2009; Rippe and Papantonis, 2025). Functionally, the high concentration of RNA polymerases II and III and associated factors, such as TFIIB and Brd4, in discrete nuclear foci should enhance transcription efficiency (Rippe and Papantonis, 2025). Moreover, recent studies on transcription compartments suggest that transcription factories are assembled by phase separation to drive gene expression, although other mechanisms of molecular nucleation and clustering may also contribute (Rippe and Papantonis, 2025).

## 5 The accessory body/“Cajal body”

### 5.1 Historical background

**Figure 5:**
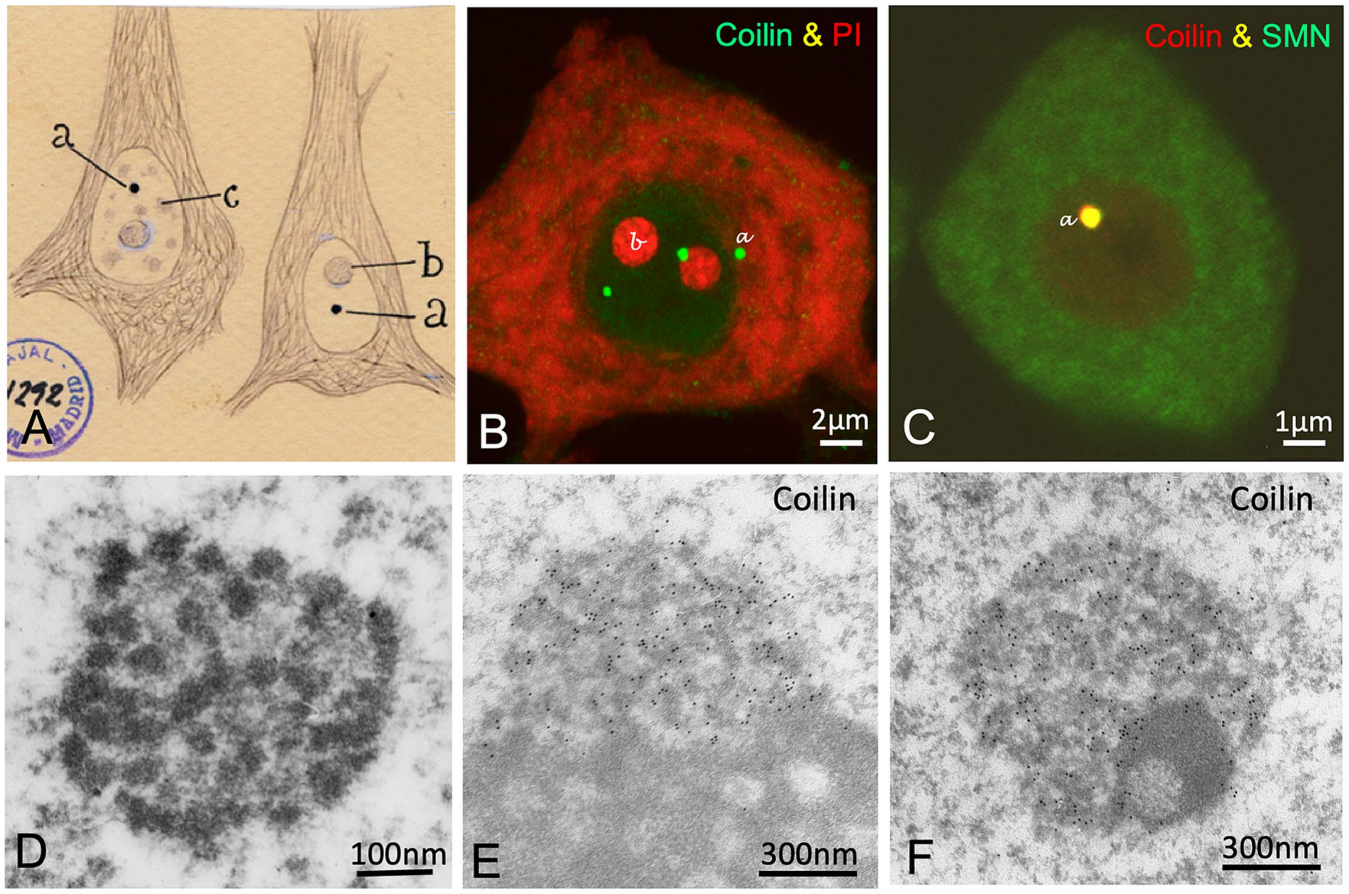
The accessory body/“Cajal body”. **(A)** Cajal’s original drawing of silver-stained accessory bodies/“Cajal bodies” in pyramidal neurons from the cerebral cortex. Note that the cytoskeleton of the neurofibrils (intermediate filaments) appears clearly defined with the silver staining. The typography of Cajal’s lettering indicates: (a) accessory bodies/“Cajal bodies,” (b) nucleolus and (c) hyaline grumes/“nuclear speckles.” (From Legado Cajal, Instituto Cajal-CSIC. Madrid, first published in Santiago Ramon y Cajal, 1910, Figure 7). **(B)** Coilin immunolabeling of accessory bodies/“Cajal bodies” (green) and counterstained with propidium iodide for rRNAs (red) in a control mouse spinal cord motor neuron (Reproduced from Tapia et al. Neurobiol Dis 2017, Figure 1A, with permission from Elsevier). **(C)** Colocalization of coilin and SMN in an accessory body/“Cajal body” of a control mouse motor neuron. (Reproduced from Lafarga et al. RNA Biol 2017, Figure 4C, with permission from Taylor & Francis). **(D)** Electron micrograph of a Cajal body in a control rat Purkinje neuron showing its substructures: the electron-dense threads and the amorphous intertwining matrix of lower electron-density (Reproduced from Lafarga et al. Anat Embryol (Berlin) 1995, Figure 10, with permission from Springer Nature). **(E,F)** Immunogold electron microscopy for coilin detection in nucleolus-associated **(E)** and nucleoplasmic free **(F)** Cajal bodies in control rat sensory ganglion neurons. Immunogold particles specifically decorate the electron-dense threads. Note the segregated electron-dense mass free of labeling in **(F)** (Reproduced from Pena et al. J Comp Neurol 2001, Figure 7A, D, with permission from Wiley-Liss INC).

In 1903, using the reduced silver nitrate procedure, Cajal discovered a round, sharply defined nuclear body in several types of neurons. This structure, approximately 0.5 μm in diameter, exhibited a strong affinity for colloidal silver (Figures 1C, 2A, 5A). He termed this structure the accessory body of the nucleolus (Cajal, 1903, 1910). Cajal also noted neuron size–dependent variation in the number of accessory bodies (from 1 to 3) and concluded that they represent a distinct nuclear structure distinguishable by morphology, size, and silver affinity from argyrophilic nucleolar spherules, hyaline grumes, the Levi basophilic grume, and micronucleoli (Figure 3B).

Cajal’s discovery was first confirmed in feline neurons by Barr’s team in 1957, who also demonstrated using the Feulgen cytochemical method that the accessory body lacks DNA (Thompson et al., 1957; Nayyar and Barr, 1968). Interestingly, in 1969, two independent groups described a new nuclear body with an identical fine structure composed of electron-dense coiled threads. In sensory ganglion neurons, Hardin’s team -familiar with Cajal’s work- identified the nuclear body as Cajal’s accessory body and retained the name (Hardin et al., 1969). Meanwhile, Monneron and Bernhard (1969) reported a similar nuclear body in hepatocytes and named it the “coiled body” based on its coiled thread configuration. Fourteen years later, using a modification of Cajal’s silver staining method for electron microscopy cytochemistry, our group definitively demonstrated that Cajal’s accessory body and Monneron and Bernhard’s “coiled body” were the same structure (Lafarga et al., 1983, 1995). The ultrastructural coiled threads of this nuclear body were specific targets of the silver reaction and appeared intensely decorated with silver grains (Figure 1E). However, it was not until 1999, at the EMBO Workshop on “Functional Organization of the Cell Nucleus” held in Prague, that Joseph Gall proposed associating Cajal’s name with the structure he discovered in 1903 (Carvalho et al., 1999; Gall et al., 1999; Gall, 2000).

The modern era of “Cajal body” (CB) research began in the 1990s with the characterization of a CB-specific protein, p80-Coilin (Andrade et al., 1991; Raška et al., 1991), which serves as a CB marker (Figures 1D,I, 5B,E–F). Subsequently, other essential CB components were identified, including spliceosomal small nuclear ribonucleoproteins (snRNPs), small nucleolar ribonucleoproteins (snoRNPs), small Cajal body-specific RNAs (scaRNAs), fibrillarin, Nopp140, WRAP53, SMN (Survival Motor Neuron), and several Gemin proteins of the SMN complex (Figure 5C) (Carmo-Fonseca et al., 1991; Liu et al., 1997; Carvalho et al., 1999; Gall, 2000; Pena et al., 2001; Darzacq et al., 2002; Cioce and Lamond, 2005; Matera and Shpargel, 2006; Staněk and Neugebauer, 2006; Machyna et al., 2013; Henriksson and Farnebo, 2015).

The ultrastructure of neuronal CBs is characterized by two internal substructures: (i) electron-dense threads with strong silver affinity, immunolabeled for coilin and fibrillarin, and (ii) an embedded amorphous matrix of lower electron density, refractory to silver staining and free of coilin and fibrillarin (Figures 1E, 5D–F) (Lafarga et al., 1995; Pena et al., 2001).

### 5.2 Functions of the Cajal body

The CB is a transcriptionally dependent nuclear structure (Lafarga et al., 1983, 2009; Carmo-Fonseca et al., 1992; Gall, 2000). For instance, the mean number of CBs increased (i) following the stimulation of supraoptic neurons, (ii) in response to elevated transcriptional demand in sensory ganglion neurons, and (iii) during high cellular proliferative states such as cancer. Conversely, CB disassembly occurs in motor neurons and Purkinje cells under cellular stress (Pena et al., 2001; Cioce and Lamond, 2005; Berciano et al., 2007; Baltanás et al., 2011b; Lafarga et al., 2017; Tapia et al., 2017).

The best-known function of the CB is the biogenesis of spliceosomal snRNPs and snoRNPs. Some of these RNPs are splicing factors required for spliceosome assembly, while others are involved in nucleolar pre-rRNA processing and additional cellular functions (Gall, 2000; Machyna et al., 2014; Matera and Wang, 2014; Arias Escayola et al., 2025). Recent advances have expanded the list of known CB components (144 identified proteins) and revealed an unexpected link between ribosomes and CB number, mediated by 60S ribosomal proteins (Arias Escayola et al., 2025).

The CB is considered a typical example of a membrane-less nuclear organelle formed through liquid–liquid phase separation (LLPS) (Zhu and Brangwynne, 2015; Hirose et al., 2023). Specifically, the architectural scaffold protein coilin and its oligomerization and post-translational modifications are critical for CB assembly (Hebert and Matera, 2000; Tucker et al., 2001, 2024; Machyna et al., 2014; Staněk, 2023; Arias Escayola et al., 2025). Recently, Basello and colleagues demonstrated that the dynamic interaction between spliceosomal snRNPs and coilin is essential for CB formation and maintenance (Basello et al., 2025). However, they also noted that CBs exhibit some, but not all, properties of nuclear condensates formed by LLPS. For example, the LLPS model alone cannot explain the CB substructures observed by electron and super-resolution microscopy (Figures 5D–F) (Raška et al., 1991; Pena et al., 2001; Basello et al., 2025).

### 5.3 Cajal bodies and spinal muscular atrophy (SMA)

Alterations in CB number and organization are directly linked to the pathophysiology of a severe human neuromuscular disorder, the spinal muscular atrophy (SMA). This monogenic disease is caused by deletions or mutations of the *SMN1* gene (Survival Motor Neuron 1), which encodes the SMN protein. SMN deficiency in SMA leads to degeneration of spinal motor neurons (Lefebvre et al., 1995). Importantly, SMN is an essential molecular constituent of the CB (Figure 5C) (Carvalho et al., 1999; Pena et al., 2001). As part of the SMN complex with Gemin proteins, SMN plays a crucial role in spliceosomal snRNP biogenesis, although its functions extend beyond this canonical role (reviewed in Matera and Wang, 2014; Bernabò et al., 2017; Chaytow et al., 2018). SMN depletion disrupts CB, nucleolar, and nuclear speckle organization in spinal motor neurons, resulting in severe dysregulation of pre-mRNA splicing, cytoplasmic mRNA depletion, and defective translational efficiency (Gabanella et al., 2007; Zhang et al., 2008; Tapia et al., 2012, 2017; Narcís et al., 2018). Furthermore, impaired WRAP53-mediated SMN trafficking to CBs, which is essential for CB biogenesis and function, has been reported in SMA patients (Henriksson and Farnebo, 2015). The link between CB dysfunction and SMA pathogenesis is further supported by studies showing restoration of normal CB number and organization in motor neurons following treatment with the antisense oligonucleotide (ASO) Nusinersen in a mouse SMA model (Berciano et al., 2007). This ASO, a splicing modulator that increases SMN protein levels, notably improves motor function (reviewed in Bennett Frank et al., 2019).

## 6 Other nuclear structures visualized by Cajal

In 1910, Cajal described additional nuclear components such as the Levi basophilic grume and the linin framework (Figure 1C). In 1896, Levi was the first to report a basophilic perinucleolar cap stained with methyl green (Levi, 1896). Cajal later confirmed and expanded on this finding, showing that the Levi basophilic grume stained intensely with toluidine blue but was refractory to silver nitrate staining (Figure 1C). Cajal pointed out that in large neurons, this nuclear body commonly appeared as a single clump oriented parallel to the axis of the pyramidal neuron, whereas smaller neurons often contained two or three smaller perinucleolar caps. The Levi basophilic grume corresponds to the “nucleolus-associated heterochromatin mass,” which is immunostained for histone H3K9me3 (Figure 1K), a marker of repressive chromatin. This structure is part of the perinucleolar compartment referred to as the “nucleolar-associated domain” (NAD) -a gene-poor, transcriptionally silent region enriched in pericentromeric heterochromatin and inactive rDNA repeats (Pontvianne et al., 2016; Hori et al., 2023; Shan et al., 2024).

Cajal also confirmed and expanded on earlier studies describing a nuclear trabecular network he termed the “linin framework” (Cajal, 1910). He proposed that this network of branched filaments connected the nucleolus to the nuclear envelope (Figure 1C). Using semithin (500 nm) resinless sections of neuronal nuclei processed by critical-point drying and analyzed via high-voltage electron microscopy, we have proposed that the linin framework corresponds to the “nucleoskeleton” (Figure 1F) (Lafarga et al., 2009; Adam, 2017). This includes the nuclear lamina, as part of the lamina-associated domains (LADs) that exert a repressive role in gene expression (Martino et al., 2023). It is now believed that the nuclear lamina, in concert with chromatin, may govern the mechanosensing properties of the nucleus (Gerace and Tapia, 2018; Stephens et al., 2017,^2019^). These properties determine how cells respond to external stimuli sensed at the plasma membrane (Miroshnikova and Wickström, 2022). This mechanotransduction mechanism converts mechanical forces into changes in gene expression (reviewed in Niethammer, 2021).

Finally, it is intriguing that Cajal (1910) and Marinesco (1909) depicted a nuclear envelope composed of a double membrane (Figure 1C), currently identified with electron microscopy (Figure 1J), even though such detail was beyond the resolution of light microscopy. We believe that the apparent double membrane seen by Cajal may have resulted from excessive dilation of the perinuclear cistern, likely due to fixative effects in his reduced silver nitrate method. It is worth mentioning that Cajal had also drawn the nucleus surrounded by two parallel lines in a prior study on the large non-neuronal cells of invertebrates (Cajal, 1898).

## 7 Conclusion

Cajal would undoubtedly be delighted to see that the nuclear structures he described in the early 20th century have been validated using modern technologies. More than a century later, the accuracy and reliability of his observations -including his quantitative estimates of certain nuclear structures-remain astonishing and inspiring. Cajal would also be pleased to know that the structures he meticulously documented are now recognized as key components in the functional organization of the nucleus and in maintaining cellular homeostasis. In recognition of his pioneering contributions to the study of the cell nucleus, Cajal’s name will forever be associated with one of its hallmark structures, the “Cajal body.”
